# Tetra­kis(μ_2_-3,4-dimethoxy­phenyl­acetato)-κ^3^ *O*,*O*′:*O*′;κ^3^ *O*:*O*:*O*′;κ^2^ *O*:*O*′;κ^2^ *O*:*O*′-bis­[(3,4-dimethoxy­phenyl­acetato-κ^2^ *O*,*O*′)(1,10-phenanthroline-κ^2^ *N*,*N*)thulium(III)]

**DOI:** 10.1107/S1600536809051435

**Published:** 2009-12-04

**Authors:** Jia-Lu Liu, Hua-Qiong Li, Guo-Liang Zhao

**Affiliations:** a Zhejiang Key Laboratory for Reactive Chemistry on Solid Surfaces, Institute of Physical Chemistry, Zhejiang Normal University, Jinhua, Zhejiang 321004, People’s Republic of China, and College of Chemistry and Life Science, Zhejiang Normal University, Jinhua 321004, Zhejiang, People’s Republic of China

## Abstract

In the title centrosymmetric dinuclear complex, [Tm_2_(C_10_H_11_O_4_)_6_(C_12_H_8_N_2_)_2_], the unique Tm^III^ ion is coordin­ated by five 3,4-dimethoxy­phenyl­acetate (DMPA) ligands and and a bis-chelating 1,10-phenanthroline (phen) ligand *via* seven O atoms and two N atoms, forming a distorted tricapped trigonal-prismatic environment. The DMPA ligands coordin­ate in the bis-chelate, bridging and bridging tridentate modes.

## Related literature

For background to the importance of coordination in magnetism, see: Yao *et al.* (2008 ▶); Fang & Zhang (2006 ▶); Li *et al.* 2008 ▶); Wang & Sevov (2008 ▶). For a related structure, see: Wang *et al.* (2008 ▶).
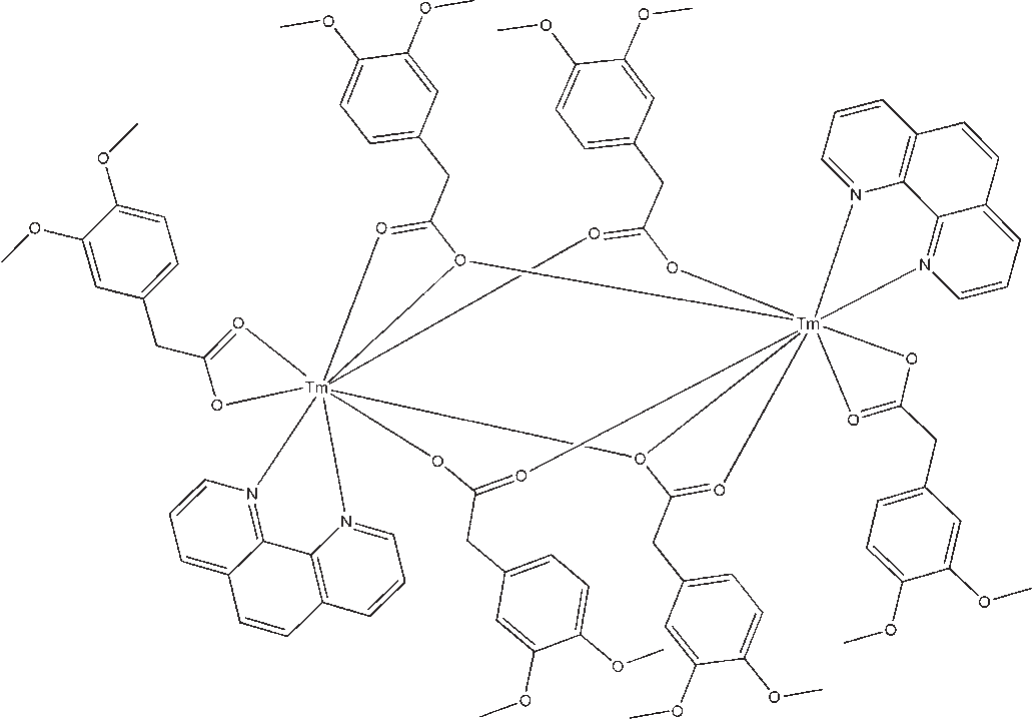


## Experimental

### 

#### Crystal data

[Tm_2_(C_10_H_11_O_4_)_6_(C_12_H_8_N_2_)_2_]
*M* *_r_* = 1869.40
Triclinic, 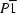

*a* = 12.3025 (1) Å
*b* = 12.3483 (2) Å
*c* = 14.5883 (2) Å
α = 91.457 (1)°
β = 103.403 (1)°
γ = 114.406 (1)°
*V* = 1944.71 (4) Å^3^
*Z* = 1
Mo *K*α radiation
μ = 2.35 mm^−1^
*T* = 296 K
0.35 × 0.11 × 0.10 mm

#### Data collection

Bruker SMART APEX diffractometer
Absorption correction: multi-scan (*SADABS*; Sheldrick, 1996 ▶) *T*_min_ = 0.747, *T*_max_ = 0.798
32353 measured reflections
6861 independent reflections
5961 reflections with *I* > 2σ(*I*)
*R*_int_ = 0.038

#### Refinement

*R*[*F* ^2^ > 2σ(*F* ^2^)] = 0.025
*wR*(*F* ^2^) = 0.058
*S* = 1.03
6861 reflections
514 parameters
H-atom parameters constrained
Δρ_max_ = 0.64 e Å^−3^
Δρ_min_ = −0.32 e Å^−3^

### 

Data collection: *SMART* (Bruker, 1997 ▶); cell refinement: *SAINT* (Bruker, 1999 ▶); data reduction: *SAINT*; program(s) used to solve structure: *SHELXS97* (Sheldrick, 2008 ▶); program(s) used to refine structure: *SHELXL97* (Sheldrick, 2008 ▶); molecular graphics: *PLATON* (Spek, 2009 ▶); software used to prepare material for publication: *SHELXL97*.

## Supplementary Material

Crystal structure: contains datablocks I, global. DOI: 10.1107/S1600536809051435/lh2956sup1.cif

Structure factors: contains datablocks I. DOI: 10.1107/S1600536809051435/lh2956Isup2.hkl

Additional supplementary materials:  crystallographic information; 3D view; checkCIF report

## Figures and Tables

**Fig. 1.**
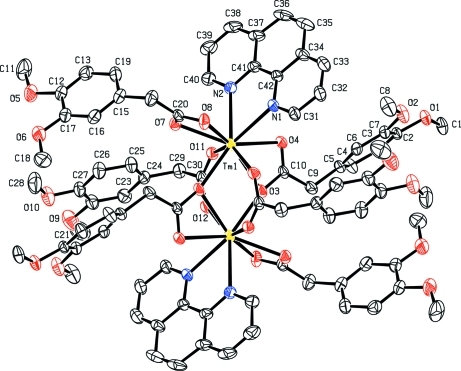
The molecular structure of the title complex. Displacement ellipsoids are drawn at the 30% probability level. Unlabeled atoms are related by the symmetry operator (-x, -y, -z).

## supplementary crystallographic information

### Comment

In recently years, there has been an increasing interest in coordination chemistry due to the increased recognition of of it's importance in magnetism (Yao, *et al.*, 2008; Fang, *et al.*, 2006; Wang, *et al.*, 2008) and this has attracted our interest (Li, *et al.*, 2008). Here we report the crystal structure of a new thulium(III) complex with the ligand 3,4-dimethoxyphenylacetate. In the title dinuclear complex (I), the unique Tm^III^ ion is coordinated by five DMPA ligands and a phen ligand via seven O atoms and two N atoms (see, Fig. 1). The Tm^III^ ion is in a distorted tricapped trigonal prismatic environment. The DMPA ligands coordinate in the bis-chelate, bridging and bridging tridentate modes.

### Experimental

All reagents and solvents were commercially available quality and without purified before using. The title compound was obtained by adding Tm_2_O_3_ (0.5 mmol), homoveratric acid (3 mmol) phen (1 mmol) dissolved in 30 ml water, sealed in a 50 ml stainless steel reactor and kept three days at temperature of 433 K. Then, the reactor was cooled to room temperature at a speed of 5 degrees per hour. Filtrate the solution, washing deposition with ethanol, then colorless histogram crystals can be attained.

### Refinement

The H atoms bonded to C atoms were positioned geometrically and refined using a riding-model approximation with C—H distances of 0.96 and 0.93 Å for aliphatic and aromatic C atoms, respectively, and *U*_iso_(H) = 1.2*U*_eq_(C) or 1.5*U*_eq_(*C*_methyl_).

### Crystal data

**Table:**

[Tm_2_(C_10_H_11_O_4_)_6_(C_12_H_8_N_2_)_2_]	*Z* = 1
*M**_r_* = 1869.40	*F*(000) = 944
Triclinic, *P*1	*D*_x_ = 1.596 Mg m^−^^3^
Hall symbol: -P 1	Mo *K*α radiation, λ = 0.71073 Å
*a* = 12.3025 (1) Å	Cell parameters from 8597 reflections
*b* = 12.3483 (2) Å	θ = 2.1–27.6°
*c* = 14.5883 (2) Å	µ = 2.35 mm^−^^1^
α = 91.457 (1)°	*T* = 296 K
β = 103.403 (1)°	Block, colourless
γ = 114.406 (1)°	0.35 × 0.11 × 0.10 mm
*V* = 1944.71 (4) Å^3^	

### Data collection

**Table:**

Bruker SMART APEX diffractometer	6861 independent reflections
Radiation source: fine-focus sealed tube	5961 reflections with *I* > 2σ(*I*)
graphite	*R*_int_ = 0.038
ω scans	θ_max_ = 25.0°, θ_min_ = 2.1°
Absorption correction: multi-scan (*SADABS*; Sheldrick, 1996)	*h* = −14→14
*T*_min_ = 0.747, *T*_max_ = 0.798	*k* = −14→14
32353 measured reflections	*l* = −17→17

### Refinement

**Table:**

Refinement on *F*^2^	Primary atom site location: structure-invariant direct methods
Least-squares matrix: full	Secondary atom site location: difference Fourier map
*R*[*F*^2^ > 2σ(*F*^2^)] = 0.025	Hydrogen site location: inferred from neighbouring sites
*wR*(*F*^2^) = 0.058	H-atom parameters constrained
*S* = 1.03	*w* = 1/[σ^2^(*F*_o_^2^) + (0.0303*P*)^2^ + 0.071*P*] where *P* = (*F*_o_^2^ + 2*F*_c_^2^)/3
6861 reflections	(Δ/σ)_max_ = 0.004
514 parameters	Δρ_max_ = 0.64 e Å^−^^3^
0 restraints	Δρ_min_ = −0.32 e Å^−^^3^

### Special details

**Table:**

Geometry. All e.s.d.'s (except the e.s.d. in the dihedral angle between two l.s. planes) are estimated using the full covariance matrix. The cell e.s.d.'s are taken into account individually in the estimation of e.s.d.'s in distances, angles and torsion angles; correlations between e.s.d.'s in cell parameters are only used when they are defined by crystal symmetry. An approximate (isotropic) treatment of cell e.s.d.'s is used for estimating e.s.d.'s involving l.s. planes.
Refinement. Refinement of *F*^2^ against ALL reflections. The weighted *R*-factor *wR* and goodness of fit *S* are based on *F*^2^, conventional *R*-factors *R* are based on *F*, with *F* set to zero for negative *F*^2^. The threshold expression of *F*^2^ > σ(*F*^2^) is used only for calculating *R*-factors(gt) *etc*. and is not relevant to the choice of reflections for refinement. *R*-factors based on *F*^2^ are statistically about twice as large as those based on *F*, and *R*- factors based on ALL data will be even larger.

### Fractional atomic coordinates and isotropic or equivalent isotropic displacement parameters (Å^2^)

**Table:**

	*x*	*y*	*z*	*U*_iso_*/*U*_eq_	
Tm1	0.175028 (11)	0.102013 (13)	0.030650 (9)	0.04097 (6)	
N1	0.3561 (2)	0.1554 (2)	0.18114 (19)	0.0465 (6)	
O1	0.3698 (2)	−0.4441 (2)	0.30577 (16)	0.0521 (6)	
C1	0.3391 (4)	−0.5686 (3)	0.2846 (3)	0.0632 (10)	
H1A	0.3974	−0.5884	0.3283	0.095*	
H1B	0.3422	−0.5848	0.2208	0.095*	
H1C	0.2572	−0.6161	0.2905	0.095*	
O2	0.3894 (2)	−0.2368 (2)	0.36761 (18)	0.0685 (7)	
N2	0.3785 (2)	0.1373 (2)	0.0008 (2)	0.0496 (7)	
C2	0.2942 (3)	−0.4026 (3)	0.2490 (2)	0.0413 (7)	
O3	0.01233 (18)	−0.0982 (2)	0.04225 (14)	0.0462 (5)	
C3	0.2150 (3)	−0.4601 (3)	0.1623 (2)	0.0512 (8)	
H3A	0.2115	−0.5318	0.1373	0.061*	
O4	0.20449 (19)	−0.0728 (2)	0.08331 (16)	0.0487 (5)	
C4	0.1394 (3)	−0.4117 (3)	0.1114 (3)	0.0581 (9)	
H4A	0.0872	−0.4512	0.0519	0.070*	
O5	0.2337 (3)	0.5128 (3)	−0.43218 (19)	0.0821 (8)	
C5	0.1398 (3)	−0.3071 (3)	0.1466 (2)	0.0460 (8)	
C6	0.2248 (3)	−0.2465 (3)	0.2326 (2)	0.0451 (8)	
H6A	0.2297	−0.1737	0.2565	0.054*	
O6	0.1019 (2)	0.5544 (3)	−0.3365 (2)	0.0800 (8)	
O7	0.21737 (19)	0.2421 (2)	−0.08600 (15)	0.0469 (5)	
C7	0.3022 (3)	−0.2915 (3)	0.2833 (2)	0.0435 (7)	
O8	0.2591 (2)	0.3120 (2)	0.06308 (15)	0.0555 (6)	
C8	0.4180 (4)	−0.1159 (4)	0.3958 (3)	0.0852 (14)	
H8A	0.4800	−0.0874	0.4556	0.128*	
H8B	0.3447	−0.1099	0.4022	0.128*	
H8C	0.4488	−0.0682	0.3486	0.128*	
C9	0.0460 (3)	−0.2642 (3)	0.0961 (3)	0.0550 (9)	
H9A	−0.0017	−0.3175	0.0369	0.066*	
H9B	−0.0107	−0.2735	0.1349	0.066*	
O9	−0.2502 (3)	−0.0455 (3)	−0.5655 (2)	0.0980 (10)	
C10	0.0931 (3)	−0.1378 (3)	0.0735 (2)	0.0416 (7)	
O10	−0.1572 (3)	0.1810 (3)	−0.5085 (2)	0.0848 (9)	
O11	0.11190 (19)	−0.0205 (2)	−0.11245 (15)	0.0470 (5)	
C11	0.3103 (5)	0.5021 (5)	−0.4862 (3)	0.1033 (16)	
H11C	0.2831	0.5165	−0.5499	0.155*	
H11D	0.3060	0.4226	−0.4875	0.155*	
H11A	0.3942	0.5598	−0.4581	0.155*	
C12	0.2587 (3)	0.4949 (3)	−0.3385 (2)	0.0576 (9)	
O12	−0.09479 (19)	−0.1233 (2)	−0.15456 (15)	0.0523 (6)	
C13	0.3461 (3)	0.4592 (3)	−0.2948 (3)	0.0625 (10)	
H13A	0.3935	0.4427	−0.3294	0.075*	
C14	0.3652 (3)	0.4470 (3)	−0.1987 (3)	0.0633 (10)	
H14A	0.4253	0.4227	−0.1693	0.076*	
C15	0.2951 (3)	0.4710 (3)	−0.1466 (2)	0.0534 (9)	
C16	0.2053 (3)	0.5051 (3)	−0.1922 (3)	0.0569 (9)	
H16A	0.1564	0.5194	−0.1580	0.068*	
C17	0.1865 (3)	0.5185 (3)	−0.2867 (3)	0.0564 (9)	
C18	0.0160 (4)	0.5660 (4)	−0.2907 (4)	0.0914 (14)	
H18A	−0.0379	0.5918	−0.3334	0.137*	
H18B	0.0602	0.6242	−0.2347	0.137*	
H18C	−0.0322	0.4899	−0.2733	0.137*	
C19	0.3135 (4)	0.4569 (3)	−0.0425 (3)	0.0640 (10)	
H19A	0.2760	0.4999	−0.0144	0.077*	
H19B	0.4015	0.4944	−0.0115	0.077*	
C20	0.2600 (3)	0.3284 (3)	−0.0220 (2)	0.0456 (8)	
C21	−0.2939 (5)	−0.1633 (5)	−0.6054 (3)	0.1053 (17)	
H21A	−0.3540	−0.1780	−0.6652	0.158*	
H21B	−0.3319	−0.2160	−0.5633	0.158*	
H21C	−0.2263	−0.1776	−0.6155	0.158*	
C22	−0.1625 (3)	−0.0060 (4)	−0.4787 (3)	0.0617 (10)	
C23	−0.1222 (3)	−0.0781 (3)	−0.4219 (3)	0.0585 (9)	
H23A	−0.1556	−0.1601	−0.4422	0.070*	
C24	−0.0336 (3)	−0.0302 (3)	−0.3362 (2)	0.0488 (8)	
C25	0.0151 (3)	0.0914 (3)	−0.3078 (2)	0.0594 (9)	
H25A	0.0751	0.1250	−0.2501	0.071*	
C26	−0.0237 (4)	0.1647 (4)	−0.3637 (3)	0.0667 (10)	
H26A	0.0107	0.2468	−0.3437	0.080*	
C27	−0.1136 (3)	0.1156 (4)	−0.4491 (2)	0.0602 (10)	
C28	−0.1058 (5)	0.3062 (5)	−0.4796 (4)	0.1056 (17)	
H28A	−0.1433	0.3422	−0.5267	0.158*	
H28B	−0.0181	0.3405	−0.4726	0.158*	
H28C	−0.1210	0.3206	−0.4199	0.158*	
C29	0.0109 (3)	−0.1085 (3)	−0.2735 (2)	0.0544 (9)	
H29A	−0.0424	−0.1925	−0.2975	0.065*	
H29B	0.0943	−0.0927	−0.2755	0.065*	
C30	0.0095 (3)	−0.0830 (3)	−0.1716 (2)	0.0459 (8)	
C31	0.3467 (3)	0.1640 (3)	0.2691 (2)	0.0534 (9)	
H31A	0.2728	0.1600	0.2779	0.064*	
C32	0.4414 (3)	0.1785 (3)	0.3499 (3)	0.0617 (10)	
H32A	0.4311	0.1860	0.4105	0.074*	
C33	0.5489 (3)	0.1816 (3)	0.3375 (3)	0.0645 (10)	
H33A	0.6129	0.1906	0.3901	0.077*	
C34	0.5632 (3)	0.1712 (3)	0.2472 (3)	0.0549 (9)	
C35	0.6728 (3)	0.1718 (3)	0.2281 (4)	0.0746 (12)	
H35A	0.7371	0.1761	0.2786	0.089*	
C36	0.6844 (3)	0.1663 (4)	0.1397 (4)	0.0761 (13)	
H36A	0.7570	0.1673	0.1302	0.091*	
C37	0.5883 (3)	0.1588 (3)	0.0593 (3)	0.0592 (10)	
C38	0.5985 (3)	0.1566 (3)	−0.0335 (3)	0.0713 (12)	
H38A	0.6712	0.1615	−0.0457	0.086*	
C39	0.5023 (4)	0.1473 (4)	−0.1062 (3)	0.0726 (12)	
H39A	0.5090	0.1484	−0.1684	0.087*	
C40	0.3922 (3)	0.1361 (3)	−0.0859 (3)	0.0600 (10)	
H40A	0.3258	0.1273	−0.1363	0.072*	
C41	0.4763 (3)	0.1515 (3)	0.0748 (3)	0.0485 (8)	
C42	0.4642 (3)	0.1595 (3)	0.1689 (2)	0.0442 (8)	

### Atomic displacement parameters (Å^2^)

**Table:**

	*U*^11^	*U*^22^	*U*^33^	*U*^12^	*U*^13^	*U*^23^
Tm1	0.04263 (9)	0.06291 (11)	0.03925 (9)	0.03882 (7)	0.01862 (6)	0.02181 (7)
N1	0.0437 (14)	0.0538 (17)	0.0553 (18)	0.0325 (13)	0.0142 (12)	0.0189 (14)
O1	0.0606 (14)	0.0536 (14)	0.0516 (14)	0.0386 (12)	0.0048 (11)	0.0131 (11)
C1	0.085 (3)	0.066 (2)	0.058 (2)	0.055 (2)	0.0112 (19)	0.0174 (19)
O2	0.0852 (18)	0.0539 (16)	0.0568 (16)	0.0380 (14)	−0.0132 (13)	0.0011 (13)
N2	0.0496 (15)	0.0606 (18)	0.0589 (18)	0.0355 (14)	0.0275 (14)	0.0227 (14)
C2	0.0425 (16)	0.052 (2)	0.0419 (18)	0.0303 (15)	0.0132 (14)	0.0166 (15)
O3	0.0473 (12)	0.0716 (15)	0.0467 (13)	0.0461 (11)	0.0211 (10)	0.0259 (11)
C3	0.062 (2)	0.057 (2)	0.048 (2)	0.0425 (18)	0.0064 (16)	0.0029 (17)
O4	0.0422 (12)	0.0622 (14)	0.0601 (14)	0.0369 (11)	0.0178 (10)	0.0243 (12)
C4	0.058 (2)	0.064 (2)	0.053 (2)	0.0375 (19)	−0.0061 (16)	0.0000 (18)
O5	0.093 (2)	0.117 (2)	0.0538 (16)	0.0547 (18)	0.0318 (15)	0.0371 (16)
C5	0.0411 (17)	0.049 (2)	0.055 (2)	0.0275 (15)	0.0089 (15)	0.0168 (16)
C6	0.0489 (18)	0.0406 (18)	0.054 (2)	0.0255 (15)	0.0155 (15)	0.0158 (16)
O6	0.0758 (18)	0.102 (2)	0.086 (2)	0.0550 (17)	0.0296 (15)	0.0426 (17)
O7	0.0538 (13)	0.0596 (15)	0.0419 (13)	0.0341 (12)	0.0197 (10)	0.0159 (12)
C7	0.0472 (17)	0.0441 (19)	0.0422 (18)	0.0235 (15)	0.0095 (14)	0.0116 (15)
O8	0.0776 (16)	0.0684 (16)	0.0387 (13)	0.0454 (13)	0.0210 (11)	0.0209 (12)
C8	0.097 (3)	0.060 (3)	0.080 (3)	0.039 (2)	−0.015 (2)	−0.010 (2)
C9	0.0455 (18)	0.055 (2)	0.070 (2)	0.0310 (17)	0.0078 (16)	0.0166 (18)
O9	0.101 (2)	0.092 (2)	0.078 (2)	0.0443 (19)	−0.0206 (18)	−0.0066 (18)
C10	0.0466 (18)	0.063 (2)	0.0347 (17)	0.0405 (17)	0.0144 (13)	0.0146 (15)
O10	0.101 (2)	0.086 (2)	0.0683 (19)	0.0523 (18)	0.0012 (16)	0.0217 (16)
O11	0.0476 (13)	0.0688 (15)	0.0452 (13)	0.0393 (12)	0.0220 (11)	0.0189 (11)
C11	0.141 (4)	0.134 (4)	0.055 (3)	0.067 (4)	0.046 (3)	0.034 (3)
C12	0.064 (2)	0.066 (2)	0.047 (2)	0.0271 (19)	0.0227 (17)	0.0229 (18)
O12	0.0468 (12)	0.0839 (17)	0.0425 (13)	0.0401 (12)	0.0180 (10)	0.0170 (12)
C13	0.067 (2)	0.073 (3)	0.063 (2)	0.035 (2)	0.0336 (19)	0.019 (2)
C14	0.069 (2)	0.074 (3)	0.059 (2)	0.042 (2)	0.0185 (19)	0.027 (2)
C15	0.066 (2)	0.048 (2)	0.048 (2)	0.0207 (17)	0.0237 (17)	0.0157 (17)
C16	0.066 (2)	0.055 (2)	0.060 (2)	0.0282 (18)	0.0294 (18)	0.0185 (18)
C17	0.061 (2)	0.055 (2)	0.060 (2)	0.0288 (18)	0.0206 (18)	0.0229 (18)
C18	0.075 (3)	0.101 (4)	0.121 (4)	0.053 (3)	0.036 (3)	0.033 (3)
C19	0.077 (3)	0.064 (2)	0.048 (2)	0.025 (2)	0.0199 (18)	0.0160 (19)
C20	0.0430 (17)	0.065 (2)	0.043 (2)	0.0352 (17)	0.0141 (14)	0.0178 (18)
C21	0.091 (3)	0.126 (5)	0.073 (3)	0.040 (3)	−0.008 (3)	−0.024 (3)
C22	0.060 (2)	0.076 (3)	0.048 (2)	0.032 (2)	0.0076 (17)	0.013 (2)
C23	0.058 (2)	0.064 (2)	0.054 (2)	0.0277 (19)	0.0145 (17)	0.0062 (19)
C24	0.0536 (19)	0.071 (3)	0.0369 (18)	0.0351 (18)	0.0225 (15)	0.0174 (17)
C25	0.070 (2)	0.066 (3)	0.041 (2)	0.033 (2)	0.0074 (17)	0.0117 (18)
C26	0.082 (3)	0.063 (2)	0.052 (2)	0.032 (2)	0.011 (2)	0.0130 (19)
C27	0.067 (2)	0.073 (3)	0.044 (2)	0.035 (2)	0.0110 (17)	0.019 (2)
C28	0.132 (4)	0.086 (4)	0.097 (4)	0.053 (3)	0.013 (3)	0.040 (3)
C29	0.066 (2)	0.075 (2)	0.046 (2)	0.046 (2)	0.0266 (16)	0.0155 (18)
C30	0.054 (2)	0.066 (2)	0.0426 (18)	0.0442 (18)	0.0218 (16)	0.0221 (17)
C31	0.054 (2)	0.064 (2)	0.054 (2)	0.0370 (18)	0.0136 (16)	0.0200 (18)
C32	0.065 (2)	0.067 (2)	0.054 (2)	0.035 (2)	0.0039 (18)	0.0174 (19)
C33	0.057 (2)	0.057 (2)	0.068 (3)	0.0271 (19)	−0.0076 (19)	0.009 (2)
C34	0.0394 (18)	0.0397 (19)	0.081 (3)	0.0204 (15)	0.0012 (17)	0.0059 (18)
C35	0.0370 (19)	0.060 (3)	0.114 (4)	0.0225 (18)	−0.004 (2)	−0.005 (3)
C36	0.0330 (19)	0.065 (3)	0.131 (4)	0.0257 (18)	0.016 (2)	−0.002 (3)
C37	0.0403 (18)	0.047 (2)	0.104 (3)	0.0265 (16)	0.0279 (19)	0.010 (2)
C38	0.049 (2)	0.063 (2)	0.120 (4)	0.0294 (19)	0.046 (2)	0.010 (2)
C39	0.073 (3)	0.076 (3)	0.097 (3)	0.041 (2)	0.055 (2)	0.023 (2)
C40	0.059 (2)	0.074 (3)	0.071 (3)	0.042 (2)	0.0354 (19)	0.024 (2)
C41	0.0388 (17)	0.0427 (19)	0.074 (2)	0.0238 (15)	0.0208 (16)	0.0148 (17)
C42	0.0380 (16)	0.0388 (18)	0.065 (2)	0.0244 (14)	0.0142 (15)	0.0163 (16)

### Geometric parameters (Å, °)

**Table:**

Tm1—O3^i^	2.2840 (18)	C12—C17	1.399 (5)
Tm1—O12^i^	2.308 (2)	O12—C30	1.258 (4)
Tm1—O11	2.317 (2)	O12—Tm1^i^	2.308 (2)
Tm1—O8	2.348 (2)	C13—C14	1.389 (5)
Tm1—O7	2.444 (2)	C13—H13A	0.9300
Tm1—O4	2.4459 (19)	C14—C15	1.382 (5)
Tm1—O3	2.497 (2)	C14—H14A	0.9300
Tm1—N2	2.502 (2)	C15—C16	1.378 (5)
Tm1—N1	2.581 (2)	C15—C19	1.508 (5)
Tm1—C20	2.750 (3)	C16—C17	1.371 (5)
Tm1—C10	2.846 (3)	C16—H16A	0.9300
Tm1—Tm1^i^	3.8506 (3)	C18—H18A	0.9600
N1—C31	1.321 (4)	C18—H18B	0.9600
N1—C42	1.363 (4)	C18—H18C	0.9600
O1—C2	1.367 (3)	C19—C20	1.514 (5)
O1—C1	1.430 (4)	C19—H19A	0.9700
C1—H1A	0.9600	C19—H19B	0.9700
C1—H1B	0.9600	C21—H21A	0.9600
C1—H1C	0.9600	C21—H21B	0.9600
O2—C7	1.367 (4)	C21—H21C	0.9600
O2—C8	1.410 (4)	C22—C27	1.379 (5)
N2—C40	1.314 (4)	C22—C23	1.392 (5)
N2—C41	1.363 (4)	C23—C24	1.378 (5)
C2—C3	1.365 (4)	C23—H23A	0.9300
C2—C7	1.404 (4)	C24—C25	1.377 (5)
O3—C10	1.280 (3)	C24—C29	1.523 (4)
O3—Tm1^i^	2.2840 (18)	C25—C26	1.388 (5)
C3—C4	1.390 (4)	C25—H25A	0.9300
C3—H3A	0.9300	C26—C27	1.383 (5)
O4—C10	1.240 (4)	C26—H26A	0.9300
C4—C5	1.375 (4)	C28—H28A	0.9600
C4—H4A	0.9300	C28—H28B	0.9600
O5—C12	1.374 (4)	C28—H28C	0.9600
O5—C11	1.403 (5)	C29—C30	1.517 (4)
C5—C6	1.387 (4)	C29—H29A	0.9700
C5—C9	1.511 (4)	C29—H29B	0.9700
C6—C7	1.376 (4)	C31—C32	1.398 (4)
C6—H6A	0.9300	C31—H31A	0.9300
O6—C17	1.362 (4)	C32—C33	1.361 (5)
O6—C18	1.425 (5)	C32—H32A	0.9300
O7—C20	1.242 (4)	C33—C34	1.378 (5)
O8—C20	1.264 (4)	C33—H33A	0.9300
C8—H8A	0.9600	C34—C42	1.418 (4)
C8—H8B	0.9600	C34—C35	1.436 (5)
C8—H8C	0.9600	C35—C36	1.333 (6)
C9—C10	1.500 (5)	C35—H35A	0.9300
C9—H9A	0.9700	C36—C37	1.430 (6)
C9—H9B	0.9700	C36—H36A	0.9300
O9—C22	1.380 (4)	C37—C38	1.388 (6)
O9—C21	1.381 (5)	C37—C41	1.414 (4)
O10—C27	1.367 (4)	C38—C39	1.356 (6)
O10—C28	1.415 (5)	C38—H38A	0.9300
O11—C30	1.259 (4)	C39—C40	1.404 (5)
C11—H11C	0.9600	C39—H39A	0.9300
C11—H11D	0.9600	C40—H40A	0.9300
C11—H11A	0.9600	C41—C42	1.419 (5)
C12—C13	1.359 (5)		
			
O3^i^—Tm1—O12^i^	75.69 (7)	O3—C10—Tm1	61.27 (16)
O3^i^—Tm1—O11	76.05 (7)	C9—C10—Tm1	178.2 (2)
O12^i^—Tm1—O11	138.51 (8)	C27—O10—C28	117.0 (3)
O3^i^—Tm1—O8	89.39 (8)	C30—O11—Tm1	135.28 (19)
O12^i^—Tm1—O8	79.23 (8)	O5—C11—H11C	109.5
O11—Tm1—O8	129.97 (7)	O5—C11—H11D	109.5
O3^i^—Tm1—O7	74.97 (7)	H11C—C11—H11D	109.5
O12^i^—Tm1—O7	124.10 (7)	O5—C11—H11A	109.5
O11—Tm1—O7	75.85 (8)	H11C—C11—H11A	109.5
O8—Tm1—O7	54.12 (8)	H11D—C11—H11A	109.5
O3^i^—Tm1—O4	124.40 (7)	C13—C12—O5	125.5 (3)
O12^i^—Tm1—O4	93.43 (7)	C13—C12—C17	119.9 (3)
O11—Tm1—O4	78.36 (7)	O5—C12—C17	114.6 (3)
O8—Tm1—O4	142.79 (8)	C30—O12—Tm1^i^	137.4 (2)
O7—Tm1—O4	142.18 (7)	C12—C13—C14	120.4 (3)
O3^i^—Tm1—O3	72.78 (7)	C12—C13—H13A	119.8
O12^i^—Tm1—O3	71.47 (8)	C14—C13—H13A	119.8
O11—Tm1—O3	71.52 (7)	C15—C14—C13	120.3 (3)
O8—Tm1—O3	148.56 (7)	C15—C14—H14A	119.9
O7—Tm1—O3	138.45 (7)	C13—C14—H14A	119.9
O4—Tm1—O3	52.42 (6)	C16—C15—C14	118.7 (3)
O3^i^—Tm1—N2	141.43 (8)	C16—C15—C19	120.3 (3)
O12^i^—Tm1—N2	139.68 (8)	C14—C15—C19	121.0 (3)
O11—Tm1—N2	78.56 (8)	C17—C16—C15	121.5 (3)
O8—Tm1—N2	85.13 (8)	C17—C16—H16A	119.2
O7—Tm1—N2	70.97 (7)	C15—C16—H16A	119.2
O4—Tm1—N2	77.18 (8)	O6—C17—C16	125.2 (3)
O3—Tm1—N2	124.89 (7)	O6—C17—C12	115.6 (3)
O3^i^—Tm1—N1	149.64 (8)	C16—C17—C12	119.2 (3)
O12^i^—Tm1—N1	75.49 (8)	O6—C18—H18A	109.5
O11—Tm1—N1	133.46 (7)	O6—C18—H18B	109.5
O8—Tm1—N1	76.06 (8)	H18A—C18—H18B	109.5
O7—Tm1—N1	114.57 (8)	O6—C18—H18C	109.5
O4—Tm1—N1	66.81 (8)	H18A—C18—H18C	109.5
O3—Tm1—N1	106.50 (7)	H18B—C18—H18C	109.5
N2—Tm1—N1	64.71 (9)	C15—C19—C20	114.9 (3)
O3^i^—Tm1—C20	81.95 (8)	C15—C19—H19A	108.5
O12^i^—Tm1—C20	102.70 (9)	C20—C19—H19A	108.5
O11—Tm1—C20	102.70 (9)	C15—C19—H19B	108.5
O8—Tm1—C20	27.28 (9)	C20—C19—H19B	108.5
O7—Tm1—C20	26.86 (8)	H19A—C19—H19B	107.5
O4—Tm1—C20	152.20 (8)	O7—C20—O8	121.0 (3)
O3—Tm1—C20	154.73 (7)	O7—C20—C19	121.7 (3)
N2—Tm1—C20	75.87 (8)	O8—C20—C19	117.3 (3)
N1—Tm1—C20	95.26 (9)	O7—C20—Tm1	62.72 (17)
O3^i^—Tm1—C10	99.16 (8)	O8—C20—Tm1	58.36 (17)
O12^i^—Tm1—C10	81.72 (8)	C19—C20—Tm1	175.2 (3)
O11—Tm1—C10	73.53 (8)	O9—C21—H21A	109.5
O8—Tm1—C10	156.48 (8)	O9—C21—H21B	109.5
O7—Tm1—C10	149.32 (8)	H21A—C21—H21B	109.5
O4—Tm1—C10	25.70 (7)	O9—C21—H21C	109.5
O3—Tm1—C10	26.72 (7)	H21A—C21—H21C	109.5
N2—Tm1—C10	100.89 (8)	H21B—C21—H21C	109.5
N1—Tm1—C10	85.93 (8)	C27—C22—O9	114.8 (3)
C20—Tm1—C10	175.58 (9)	C27—C22—C23	119.8 (3)
O3^i^—Tm1—Tm1^i^	38.27 (5)	O9—C22—C23	125.4 (4)
O12^i^—Tm1—Tm1^i^	69.35 (5)	C24—C23—C22	121.2 (4)
O11—Tm1—Tm1^i^	69.60 (5)	C24—C23—H23A	119.4
O8—Tm1—Tm1^i^	123.15 (6)	C22—C23—H23A	119.4
O7—Tm1—Tm1^i^	109.36 (5)	C25—C24—C23	118.5 (3)
O4—Tm1—Tm1^i^	86.54 (5)	C25—C24—C29	120.0 (3)
O3—Tm1—Tm1^i^	34.51 (4)	C23—C24—C29	121.5 (3)
N2—Tm1—Tm1^i^	146.58 (7)	C24—C25—C26	121.1 (3)
N1—Tm1—Tm1^i^	134.10 (6)	C24—C25—H25A	119.4
C20—Tm1—Tm1^i^	120.22 (6)	C26—C25—H25A	119.4
C10—Tm1—Tm1^i^	61.01 (6)	C27—C26—C25	119.9 (4)
C31—N1—C42	117.6 (3)	C27—C26—H26A	120.0
C31—N1—Tm1	124.5 (2)	C25—C26—H26A	120.0
C42—N1—Tm1	117.2 (2)	O10—C27—C22	116.6 (3)
C2—O1—C1	116.1 (2)	O10—C27—C26	123.9 (4)
O1—C1—H1A	109.5	C22—C27—C26	119.5 (3)
O1—C1—H1B	109.5	O10—C28—H28A	109.5
H1A—C1—H1B	109.5	O10—C28—H28B	109.5
O1—C1—H1C	109.5	H28A—C28—H28B	109.5
H1A—C1—H1C	109.5	O10—C28—H28C	109.5
H1B—C1—H1C	109.5	H28A—C28—H28C	109.5
C7—O2—C8	117.0 (3)	H28B—C28—H28C	109.5
C40—N2—C41	118.4 (3)	C30—C29—C24	110.6 (3)
C40—N2—Tm1	121.5 (2)	C30—C29—H29A	109.5
C41—N2—Tm1	120.0 (2)	C24—C29—H29A	109.5
C3—C2—O1	124.8 (3)	C30—C29—H29B	109.5
C3—C2—C7	118.9 (3)	C24—C29—H29B	109.5
O1—C2—C7	116.4 (3)	H29A—C29—H29B	108.1
C10—O3—Tm1^i^	158.6 (2)	O12—C30—O11	125.8 (3)
C10—O3—Tm1	92.01 (18)	O12—C30—C29	116.7 (3)
Tm1^i^—O3—Tm1	107.22 (7)	O11—C30—C29	117.4 (3)
C2—C3—C4	120.2 (3)	N1—C31—C32	123.9 (3)
C2—C3—H3A	119.9	N1—C31—H31A	118.0
C4—C3—H3A	119.9	C32—C31—H31A	118.0
C10—O4—Tm1	95.47 (17)	C33—C32—C31	118.4 (4)
C5—C4—C3	121.8 (3)	C33—C32—H32A	120.8
C5—C4—H4A	119.1	C31—C32—H32A	120.8
C3—C4—H4A	119.1	C32—C33—C34	120.3 (3)
C12—O5—C11	117.8 (3)	C32—C33—H33A	119.9
C4—C5—C6	117.4 (3)	C34—C33—H33A	119.9
C4—C5—C9	121.0 (3)	C33—C34—C42	118.1 (3)
C6—C5—C9	121.5 (3)	C33—C34—C35	123.7 (4)
C7—C6—C5	121.7 (3)	C42—C34—C35	118.2 (4)
C7—C6—H6A	119.2	C36—C35—C34	121.5 (4)
C5—C6—H6A	119.2	C36—C35—H35A	119.2
C17—O6—C18	118.0 (3)	C34—C35—H35A	119.2
C20—O7—Tm1	90.42 (18)	C35—C36—C37	121.8 (3)
O2—C7—C6	125.3 (3)	C35—C36—H36A	119.1
O2—C7—C2	114.9 (3)	C37—C36—H36A	119.1
C6—C7—C2	119.8 (3)	C38—C37—C41	118.3 (3)
C20—O8—Tm1	94.4 (2)	C38—C37—C36	123.4 (3)
O2—C8—H8A	109.5	C41—C37—C36	118.3 (4)
O2—C8—H8B	109.5	C39—C38—C37	119.8 (3)
H8A—C8—H8B	109.5	C39—C38—H38A	120.1
O2—C8—H8C	109.5	C37—C38—H38A	120.1
H8A—C8—H8C	109.5	C38—C39—C40	118.9 (4)
H8B—C8—H8C	109.5	C38—C39—H39A	120.6
C10—C9—C5	118.0 (3)	C40—C39—H39A	120.6
C10—C9—H9A	107.8	N2—C40—C39	123.2 (4)
C5—C9—H9A	107.8	N2—C40—H40A	118.4
C10—C9—H9B	107.8	C39—C40—H40A	118.4
C5—C9—H9B	107.8	N2—C41—C37	121.3 (3)
H9A—C9—H9B	107.1	N2—C41—C42	118.6 (3)
C22—O9—C21	118.6 (4)	C37—C41—C42	120.1 (3)
O4—C10—O3	120.1 (3)	N1—C42—C34	121.7 (3)
O4—C10—C9	122.9 (3)	N1—C42—C41	118.4 (3)
O3—C10—C9	117.0 (3)	C34—C42—C41	119.9 (3)
O4—C10—Tm1	58.82 (15)		
			
O3^i^—Tm1—N1—C31	25.7 (3)	N2—Tm1—C10—O4	23.0 (2)
O12^i^—Tm1—N1—C31	6.9 (2)	N1—Tm1—C10—O4	−40.31 (18)
O11—Tm1—N1—C31	−138.2 (2)	Tm1^i^—Tm1—C10—O4	172.9 (2)
O8—Tm1—N1—C31	89.2 (3)	O3^i^—Tm1—C10—O3	−9.1 (2)
O7—Tm1—N1—C31	128.1 (2)	O12^i^—Tm1—C10—O3	64.85 (16)
O4—Tm1—N1—C31	−93.3 (3)	O11—Tm1—C10—O3	−81.50 (16)
O3—Tm1—N1—C31	−58.3 (3)	O8—Tm1—C10—O3	101.0 (2)
N2—Tm1—N1—C31	−179.6 (3)	O7—Tm1—C10—O3	−85.0 (2)
C20—Tm1—N1—C31	108.7 (3)	O4—Tm1—C10—O3	−178.9 (3)
C10—Tm1—N1—C31	−75.6 (3)	N2—Tm1—C10—O3	−155.98 (16)
Tm1^i^—Tm1—N1—C31	−33.8 (3)	N1—Tm1—C10—O3	140.75 (17)
O3^i^—Tm1—N1—C42	−163.31 (18)	Tm1^i^—Tm1—C10—O3	−6.08 (13)
O12^i^—Tm1—N1—C42	178.0 (2)	O3^i^—Tm1—O11—C30	−22.7 (3)
O11—Tm1—N1—C42	32.8 (2)	O12^i^—Tm1—O11—C30	25.6 (3)
O8—Tm1—N1—C42	−99.8 (2)	O8—Tm1—O11—C30	−99.8 (3)
O7—Tm1—N1—C42	−60.8 (2)	O7—Tm1—O11—C30	−100.4 (3)
O4—Tm1—N1—C42	77.7 (2)	O4—Tm1—O11—C30	107.5 (3)
O3—Tm1—N1—C42	112.7 (2)	O3—Tm1—O11—C30	53.5 (3)
N2—Tm1—N1—C42	−8.6 (2)	N2—Tm1—O11—C30	−173.4 (3)
C20—Tm1—N1—C42	−80.3 (2)	N1—Tm1—O11—C30	149.0 (3)
C10—Tm1—N1—C42	95.5 (2)	C20—Tm1—O11—C30	−100.9 (3)
Tm1^i^—Tm1—N1—C42	137.26 (18)	C10—Tm1—O11—C30	81.5 (3)
O3^i^—Tm1—N2—C40	−16.0 (3)	Tm1^i^—Tm1—O11—C30	16.9 (3)
O12^i^—Tm1—N2—C40	−165.9 (2)	C11—O5—C12—C13	−5.0 (6)
O11—Tm1—N2—C40	33.5 (3)	C11—O5—C12—C17	174.0 (4)
O8—Tm1—N2—C40	−98.9 (3)	O5—C12—C13—C14	178.4 (4)
O7—Tm1—N2—C40	−45.3 (3)	C17—C12—C13—C14	−0.7 (6)
O4—Tm1—N2—C40	114.0 (3)	C12—C13—C14—C15	0.1 (6)
O3—Tm1—N2—C40	91.2 (3)	C13—C14—C15—C16	1.0 (6)
N1—Tm1—N2—C40	−175.8 (3)	C13—C14—C15—C19	179.3 (3)
C20—Tm1—N2—C40	−72.9 (3)	C14—C15—C16—C17	−1.6 (5)
C10—Tm1—N2—C40	104.1 (3)	C19—C15—C16—C17	−179.9 (3)
Tm1^i^—Tm1—N2—C40	51.3 (3)	C18—O6—C17—C16	−7.7 (6)
O3^i^—Tm1—N2—C41	167.8 (2)	C18—O6—C17—C12	172.8 (4)
O12^i^—Tm1—N2—C41	17.9 (3)	C15—C16—C17—O6	−178.4 (3)
O11—Tm1—N2—C41	−142.6 (2)	C15—C16—C17—C12	1.1 (5)
O8—Tm1—N2—C41	84.9 (2)	C13—C12—C17—O6	179.7 (3)
O7—Tm1—N2—C41	138.5 (2)	O5—C12—C17—O6	0.5 (5)
O4—Tm1—N2—C41	−62.1 (2)	C13—C12—C17—C16	0.1 (6)
O3—Tm1—N2—C41	−85.0 (2)	O5—C12—C17—C16	−179.1 (3)
N1—Tm1—N2—C41	8.0 (2)	C16—C15—C19—C20	102.8 (4)
C20—Tm1—N2—C41	111.0 (2)	C14—C15—C19—C20	−75.4 (4)
C10—Tm1—N2—C41	−72.1 (2)	Tm1—O7—C20—O8	−3.0 (3)
Tm1^i^—Tm1—N2—C41	−124.9 (2)	Tm1—O7—C20—C19	177.8 (3)
C1—O1—C2—C3	−17.4 (4)	Tm1—O8—C20—O7	3.1 (3)
C1—O1—C2—C7	163.7 (3)	Tm1—O8—C20—C19	−177.6 (2)
O3^i^—Tm1—O3—C10	170.6 (2)	C15—C19—C20—O8	−171.4 (3)
O12^i^—Tm1—O3—C10	−109.14 (17)	O3^i^—Tm1—C20—O7	−72.51 (16)
O11—Tm1—O3—C10	89.95 (17)	O12^i^—Tm1—C20—O7	−145.79 (16)
O8—Tm1—O3—C10	−131.31 (18)	O11—Tm1—C20—O7	1.14 (17)
O7—Tm1—O3—C10	129.97 (17)	O8—Tm1—C20—O7	−177.0 (3)
O4—Tm1—O3—C10	0.58 (16)	O4—Tm1—C20—O7	90.2 (2)
N2—Tm1—O3—C10	29.2 (2)	O3—Tm1—C20—O7	−72.4 (3)
N1—Tm1—O3—C10	−41.16 (17)	N2—Tm1—C20—O7	75.68 (17)
C20—Tm1—O3—C10	170.5 (2)	N1—Tm1—C20—O7	137.93 (16)
Tm1^i^—Tm1—O3—C10	170.6 (2)	Tm1^i^—Tm1—C20—O7	−72.47 (17)
O3^i^—Tm1—O3—Tm1^i^	0.0	O3^i^—Tm1—C20—O8	104.51 (17)
O12^i^—Tm1—O3—Tm1^i^	80.27 (8)	O12^i^—Tm1—C20—O8	31.24 (18)
O11—Tm1—O3—Tm1^i^	−80.64 (8)	O11—Tm1—C20—O8	178.16 (16)
O8—Tm1—O3—Tm1^i^	58.10 (16)	O7—Tm1—C20—O8	177.0 (3)
O7—Tm1—O3—Tm1^i^	−40.62 (13)	O4—Tm1—C20—O8	−92.7 (2)
O4—Tm1—O3—Tm1^i^	−170.01 (13)	O3—Tm1—C20—O8	104.6 (2)
N2—Tm1—O3—Tm1^i^	−141.43 (9)	N2—Tm1—C20—O8	−107.29 (18)
N1—Tm1—O3—Tm1^i^	148.25 (9)	N1—Tm1—C20—O8	−45.04 (18)
C20—Tm1—O3—Tm1^i^	−0.1 (2)	Tm1^i^—Tm1—C20—O8	104.56 (17)
C10—Tm1—O3—Tm1^i^	−170.6 (2)	C21—O9—C22—C27	173.0 (4)
O1—C2—C3—C4	178.1 (3)	C21—O9—C22—C23	−7.0 (6)
C7—C2—C3—C4	−3.1 (5)	C27—C22—C23—C24	−0.2 (6)
O3^i^—Tm1—O4—C10	−12.2 (2)	O9—C22—C23—C24	179.8 (4)
O12^i^—Tm1—O4—C10	62.79 (19)	C22—C23—C24—C25	−0.3 (5)
O11—Tm1—O4—C10	−76.13 (18)	C22—C23—C24—C29	179.9 (3)
O8—Tm1—O4—C10	139.46 (18)	C23—C24—C25—C26	0.1 (5)
O7—Tm1—O4—C10	−123.88 (19)	C29—C24—C25—C26	179.9 (3)
O3—Tm1—O4—C10	−0.60 (16)	C24—C25—C26—C27	0.6 (6)
N2—Tm1—O4—C10	−156.9 (2)	C28—O10—C27—C22	−178.8 (4)
N1—Tm1—O4—C10	135.4 (2)	C28—O10—C27—C26	0.1 (6)
C20—Tm1—O4—C10	−171.3 (2)	O9—C22—C27—O10	−0.2 (5)
Tm1^i^—Tm1—O4—C10	−6.26 (18)	C23—C22—C27—O10	179.8 (3)
C2—C3—C4—C5	−1.3 (5)	O9—C22—C27—C26	−179.1 (4)
C3—C4—C5—C6	4.3 (5)	C23—C22—C27—C26	0.9 (6)
C3—C4—C5—C9	−172.3 (3)	C25—C26—C27—O10	−179.9 (4)
C4—C5—C6—C7	−3.0 (5)	C25—C26—C27—C22	−1.1 (6)
C9—C5—C6—C7	173.6 (3)	C25—C24—C29—C30	48.9 (4)
O3^i^—Tm1—O7—C20	102.08 (17)	C23—C24—C29—C30	−131.3 (3)
O12^i^—Tm1—O7—C20	41.48 (19)	Tm1^i^—O12—C30—O11	8.3 (5)
O11—Tm1—O7—C20	−178.85 (17)	Tm1^i^—O12—C30—C29	−169.3 (2)
O8—Tm1—O7—C20	1.68 (16)	Tm1—O11—C30—O12	−22.4 (5)
O4—Tm1—O7—C20	−130.47 (17)	Tm1—O11—C30—C29	155.2 (2)
O3—Tm1—O7—C20	142.17 (16)	C24—C29—C30—O12	74.1 (4)
N2—Tm1—O7—C20	−96.29 (18)	C24—C29—C30—O11	−103.7 (3)
N1—Tm1—O7—C20	−47.19 (18)	C42—N1—C31—C32	0.9 (5)
C10—Tm1—O7—C20	−175.35 (16)	Tm1—N1—C31—C32	171.9 (3)
Tm1^i^—Tm1—O7—C20	119.15 (16)	N1—C31—C32—C33	−1.5 (6)
C8—O2—C7—C6	−12.4 (5)	C31—C32—C33—C34	0.4 (5)
C8—O2—C7—C2	168.0 (3)	C32—C33—C34—C42	1.0 (5)
C5—C6—C7—O2	179.1 (3)	C32—C33—C34—C35	−179.0 (3)
C5—C6—C7—C2	−1.2 (5)	C33—C34—C35—C36	−177.3 (4)
C3—C2—C7—O2	−176.0 (3)	C42—C34—C35—C36	2.7 (6)
O1—C2—C7—O2	2.9 (4)	C34—C35—C36—C37	−0.4 (6)
C3—C2—C7—C6	4.3 (4)	C35—C36—C37—C38	178.0 (4)
O1—C2—C7—C6	−176.8 (3)	C35—C36—C37—C41	−3.0 (6)
O3^i^—Tm1—O8—C20	−73.46 (17)	C41—C37—C38—C39	−0.1 (5)
O12^i^—Tm1—O8—C20	−149.01 (18)	C36—C37—C38—C39	178.8 (4)
O11—Tm1—O8—C20	−2.3 (2)	C37—C38—C39—C40	−2.1 (6)
O7—Tm1—O8—C20	−1.66 (16)	C41—N2—C40—C39	0.7 (5)
O4—Tm1—O8—C20	129.61 (17)	Tm1—N2—C40—C39	−175.5 (3)
O3—Tm1—O8—C20	−127.64 (18)	C38—C39—C40—N2	1.9 (6)
N2—Tm1—O8—C20	68.32 (18)	C40—N2—C41—C37	−3.0 (5)
N1—Tm1—O8—C20	133.45 (18)	Tm1—N2—C41—C37	173.2 (2)
C10—Tm1—O8—C20	174.55 (18)	C40—N2—C41—C42	176.6 (3)
Tm1^i^—Tm1—O8—C20	−92.58 (17)	Tm1—N2—C41—C42	−7.1 (4)
C4—C5—C9—C10	−128.0 (4)	C38—C37—C41—N2	2.8 (5)
C6—C5—C9—C10	55.6 (5)	C36—C37—C41—N2	−176.2 (3)
Tm1—O4—C10—O3	1.1 (3)	C38—C37—C41—C42	−176.8 (3)
Tm1—O4—C10—C9	−179.4 (3)	C36—C37—C41—C42	4.1 (5)
Tm1^i^—O3—C10—O4	153.6 (4)	C31—N1—C42—C34	0.6 (4)
Tm1—O3—C10—O4	−1.1 (3)	Tm1—N1—C42—C34	−171.0 (2)
Tm1^i^—O3—C10—C9	−26.0 (7)	C31—N1—C42—C41	−179.5 (3)
Tm1—O3—C10—C9	179.4 (3)	Tm1—N1—C42—C41	8.9 (4)
Tm1^i^—O3—C10—Tm1	154.6 (5)	C33—C34—C42—N1	−1.6 (5)
C5—C9—C10—O4	9.4 (5)	C35—C34—C42—N1	178.4 (3)
C5—C9—C10—O3	−171.1 (3)	C33—C34—C42—C41	178.5 (3)
O3^i^—Tm1—C10—O4	169.83 (18)	C35—C34—C42—C41	−1.5 (5)
O12^i^—Tm1—C10—O4	−116.22 (19)	N2—C41—C42—N1	−1.5 (4)
O11—Tm1—C10—O4	97.43 (18)	C37—C41—C42—N1	178.2 (3)
O8—Tm1—C10—O4	−80.1 (3)	N2—C41—C42—C34	178.4 (3)
O7—Tm1—C10—O4	93.9 (2)	C37—C41—C42—C34	−1.9 (5)
O3—Tm1—C10—O4	178.9 (3)		

Symmetry codes: (i) −*x*, −*y*, −*z*.
